# Subcutaneous Apomorphine Infusion Initiation Is Associated with Impulse Control Disorder Attenuation in Advanced Parkinson's Disease Patients: Insights from the French NS‐Park Cohort

**DOI:** 10.1002/mdc3.70240

**Published:** 2025-07-17

**Authors:** Clément Desjardins, Paulo André Dias Bastos, Aymeric Lanore, Christine Brefel‐Courbon, Isabelle Benatru, Caroline Giordana, Anne Doe de Maindreville, Giovanni Castelnovo, Philippe Remy, Luc Defebvre, Claire Thiriez, Stéphane Prange, Jean‐Luc Houeto, Alexandra Samier Foubert, Fabienne Ory‐Magne, Raquel Pinhero Barbosa, Nathalie Bertille, Jean‐Christophe Corvol, Olivier Rascol, Margherita Fabbri, the NS‐Part Cohort study group, Jean‐Christophe Corvol, Olivier Rascol, Margherita Fabbri, Wassilios Meissner, Stéphane Thobois, Luc Defebvre, Helene Esperou, Florence Tubach, Yann De Rycke, Layidé Roufai, Dalila Ouazib, Sandra Zarrad, Fatma Khelifi, Florence Tubach, Yann De Rycke, Nathalie Bertille, Vanessa Rousseau, Samuel Tessier, Margherita Fabbri, David Tavel, Dounya Metdaoui, Avigaelle Abitbol, Mathias Antunes, Alexis Brice, Suzanne Lesage, Christelle Tessson, Fanny Casse, Mélanie Ferrien, Guillaume Cogan, Lisa Welment, Thierry Moulin, Wassilios Meissner, Claire Thiriez, Stéphane Thobois, Christian Geny, Solène Frismand, Anne‐Gaëlle Corbille, Caroline Giordana, Giovanni Castelnovo, Isabelle Benatru, Sophie Drapier, David Maltete, Christine Tranchant, Olivier Rascol, Mickael Aubignat, Marie Mongin, Matthieu Bereau, Alexandra Foubert‐Samier, Brice Laurens, Sylvain Vergnet, Thomas Boraud, David Bendetowicz, Jade Sarrabere, Dominique Guehl, Pierre Burbaud, Paul‐Alexandre Pfeiffer, Gilles Defer, Bérengère Debilly, Philippe Derost, Alice Dormeuil, Aimée Petit, Alban Gravier, Vincent Schneider, Lucie Garnier, Nicolas Carriere, Olivier Colin, Philippe Couratier, Chloé Laurencin, Stephane Prange, Paul Jaulent, Bruno Plus, Helene Gervais‐Bernard, Alexandre Eusebio, Frédérique Fluchere, Stephan Grimaldi, Valentin Mira, Tatiana Witjas, Mahmoud Charif, Ophelie Forster, Alix Durand, Pauline Prin, Lucie Hopes, Amory Jardel, Salome Puisieux, Guillemette Clement, Philippe Damier, Tiphaine Rouaud, Pascal Derkinderen, Arthur Lionnet, Adrien De Guilhem De Lataillade, Cosmin Alecu, Charlotte Heraud, Marie De Verdal, Graziella Mangone, Sara Sambin, Aymeric Lanore, Thomas Courtin, Louise‐Laure Mariani, Fouad Khoury, Poornima Menon, Florence Cormier‐Dequaire, Emmanuel Flamand‐Roze, David Grabli, Elodie Hainque, Marie Vidhaillet, Aurélie Meneret, Cécile Delorme, Cendrine Foucard, Florian Von Raison, Alexis Elbaz, Andreas Hartmann, Vincent Leclercq, Theodore Soulier, Daniel Torres, Giulia Coarelli, Giorgia Querin, Fabien Hauw, Margaux Dunoyer, Solène Ansquer, Frederique Leh, Marion Leclercq, Simon Lamy, Guillaume Carey, Guillaume Costentin, Clemence Hardy, Mathieu Anheim, Ouhaid Lagha Boukbiza, Thomas Wirth, Jimmy Voirin, Marie Des Neiges Santin, Thomas Bogdan, Margherita Fabbri, Christine Brefel Courbon Ory‐Magne, Clemence Leung, Hélène Catala, Gabrielle Sill, Raquel Pinheiro Barbosa, Lucy Famer, Lucie Braccagni, Hiba Sifaoui, Juliette Palisson, Kenza Benrahmoune Idrissi, Astrid Causel, Lydie Romeo, Constance Bissessur, Audace Cure‐Martin, Charline Compagne, Sandrine Dupouy, Sandrine Villars, Wei‐Ho Lai, Rachida Bari, Damien Chevanne, Elodie Durand, Isabelle Rieu, Stephane Bernard, Corinne Garsault, Nathalie Meunier, Alexia Cresson, Marine Sgard, Marie Dreano, Justine Montillot, Renaud Massart, Pascale Grebent, Pierre Pelissier, Valérie Santraine, Thomas Gaudin, Pierre Boutet, Cécile Thuilier, Coralie Chalot, Céline Prevost, Hélène Videaud, Justine Picut, Christian Tarrade, Catherine Caire, Hélène Merle, Elise Metereau, Mathilde Millot, Chloe Bernardi, Emilie Favre, Adelaide Jaulent, Laura Mundler, Blandine Dufresne, Eve Benchetrit, Valérie Driss, Alexia Arifi, Maura Rodrigues, Lili Le Monnier, Nathalie Dumont, Virginie Bablon, Régis Frenais, Caroline Herve, Christelle Guimber, Vanessa Ferrier, Elodie David, Christina Faroul, Leslie Fra, Elsa Foucaran, Fatima‐Ezzahra Ennaji, Jeremy Bonetto, Ryad Ladghem‐Chikouche, Le Mickael, Sophie Liot, Sonia Messar, Hamza Salah, Amelie Bernardo, Naoual Serari, Emilie Rabois, Carole David, Zoe Fournier, Margaux Bonnaire‐Verdier, Elise Chevaillier, Françoise Kestens, Rozenn Gourhan, Sandra Lopez‐Alfaro, Jean‐François Houvenaghel, Mélanie Alexandre, Christine Bourdonnais, Linda Vernon, Ahmed Boumediene, Hugo Rummel, Céline Julie, Nadine Longato, Célie Phillipps, Anne Claire Andries‐Ros, Stéphanie Bras, Estelle Harroch, Claudia Gillet, Yoan Herades, Eva Camgrand

**Affiliations:** ^1^ Department of Neurology Rothschild Foundation Hospital Paris France; ^2^ Department of Clinical Pharmacology and Neurosciences, Toulouse Parkinson Expert Centre, Toulouse NeuroToul Center of Excellence in Neurodegeneration (COEN), French NS‐Park/F‐CRIN Network University of Toulouse 3, CHU of Toulouse, INSERM Toulouse France; ^3^ Assistance Publique Hôpitaux de Paris, Department of Neurology CIC Neurosciences Paris France; ^4^ Department of Neurology Poitiers University Hospital, INSERM, CHU de Poitiers Poitiers France; ^5^ Department of Neurology Centre Hospitalier Universitaire de Nice, Université Côte d'Azur Nice France; ^6^ Department of Neurology Hôpital Maison Blanche Reims France; ^7^ Department of Neurology University Hospital of Nîmes Nîmes France; ^8^ Centre Expert Parkinson, Neurologie, CHU Henri Mondor, AP‐HP, Equipe 01‐NPI, IMRB INSERM et Faculté de Santé Université Paris‐Est Créteil, Créteil, et Ecole Normale Supérieure Paris France; ^9^ Movement Disorders Department Lille University, Inserm 1172 Lille France; ^10^ Department of Neurology and Parkinson Expert Centre Caen University‐Hospital Caen France; ^11^ Univ Lyon, Hospices Civils de Lyon, Pierre Wertheimer Neurological Hospital, Expert Parkinson Center Université Claude‐Bernard Lyon 1 Bron France; ^12^ Department of Neurology Limoges University Hospital Limoges Cedex France; ^13^ Institut des Maladies Neurodégénératives University Bordeaux, CNRS, IMN, UMR5293 Bordeaux France; ^14^ CHU Bordeaux Service de Neurologie des Maladies Neurodégénératives, IMNc, CRMR AMS, NS‐Park/FCRIN Network Bordeaux France; ^15^ INSERM, Institut Pierre Louis d'Epidémiologie et de Santé Publique, équipe PEPITES, AP‐HP, Hôpital Pitié Salpêtrière, Département de Santé Publique Sorbonne Université Paris France; ^16^ Institut du Cerveau, Paris Brain Institute, ICM, Inserm, CNRS Sorbonne Université Paris France; ^17^ Department of Neurology Assistance Publique Hôpitaux de Paris, CIC Neurosciences, Hôpital Pitié‐Salpêtrière Paris France

**Keywords:** Parkinson's disease, impulse control disorders, continuous‐subcutaneous apomorphine infusion, cohort

## Abstract

**Background:**

Impulse control disorders (ICD) are common non‐motor complications in Parkinson's disease (PD), particularly in patients receiving oral dopamine agonists (DA). Continuous subcutaneous apomorphine infusion (CSAI) is a device‐aided therapy for advanced PD, but its effects on ICD remain underexplored in real‐world settings.

**Objectives:**

To assess the impact of CSAI initiation on ICD prevalence and severity in a large real‐world PD cohort and to compare ICD evolution in CSAI‐treated patients versus orally‐treated controls.

**Methods:**

We analyzed data from the national prospective observational NS‐Park cohort, selecting patients with documented ICD status before and after CSAI initiation. Changes in ICD prevalence and severity based on the MDS‐UPDRS sub‐item 1.6 were assessed using paired statistical tests, with additional sensitivity analyses based on time‐restricted sub‐cohorts (considering 60‐, 24‐ and 12‐months follow‐up). A matched case–control analysis and a propensity score matching were used to compare CSAI‐treated patients to orally‐treated PD patients.

**Results:**

149 patients were included in the analysis. Before CSAI initiation, slight and mild/severe ICDs were present in 17% and 5% of the patients, respectively. After CSAI starting, ICD prevalence significantly decreased from 22% to 13%, (*P* = 0.003). These improvements were consistent across different time windows, despite an overall increase in DA levodopa‐equivalent dose, with no associated mood worsening (up to 24‐month follow‐up). CSAI was associated with longitudinal ICD reduction, contrasting with the stable or worsening ICD trajectory in orally‐treated controls, though trajectories were not statistically different.

**Conclusions:**

The presented findings of our real‐life cohort suggest that ICD tend to improve following CSAI initiation in patients with PD, likely due to a reduction of oral DA or the effect of continuous dopaminergic stimulation provided by the pump. While this observation is clinically relevant, it should be interpreted with caution given the study's observational design and the limitations inherent to using MDS‐UPDRS sub‐items for ICD assessment.

Continuous subcutaneous apomorphine infusion (CSAI) is a device‐aided therapy (DAT) designed to address motor complications in advanced Parkinson's disease (PD).^1^ Its effect on dyskinesia and motor complications has been proved by one large, randomized trial^1^ and multiple observational studies.^2^, ^3^ Nevertheless the non‐motor impact of CSAI, particularly on impulse control disorders (ICD), remains insufficiently studied, with no formal agreement on non‐motor symptoms patients’ profile for DAT selection.^4^, ^5^

Oral dopamine agonist (DA) therapy in PD is associated with a 2 to 3.5 times higher risk of developing ICD, as documented in prior large‐scale studies.^6^ These behavioral disturbances, characterized by compulsive and impulsive urges, encompass pathological gambling, hypersexuality, compulsive shopping, binge eating, punding, and dopamine dysregulation syndrome. The prevalence of ICD in PD is approximately 6–7%,^7^ but it can rise to 17% in patients treated with oral DA.^8^ However, data on the prevalence of ICDs among patients treated with CSAI remain scarce.^9^ While preliminary studies involving small cohorts suggest a lower prevalence of ICD in patients treated with CSAI compared to those receiving oral DA or other dopaminergic therapies,^10^, ^11^, ^12^, ^13^ the limited sample sizes necessitate further investigation to validate these findings in larger cohorts. Furthermore, some of these studies indicate that CSAI may stabilize ICD symptoms following its initiation as a second‐line treatment.^12^, ^13^, ^14^ CSAI has also been suggested as a safer alternative to oral DA for patients with pre‐existing ICD, with some authors advocating its use in such cases.^15^ Despite these promising findings, existing studies face notable limitations, particularly small sample sizes. Moreover, ICD have also been reported as a side effect of CSAI,^1^, ^3^, ^16^, ^17^, ^18^ even in patients without a prior history of ICD.^10^, ^12^, ^19^ In rare cases, these adverse effects have necessitated the discontinuation of therapy.^11^, ^20^, ^21^, ^22^

Our study aimed to evaluate the impact of CSAI on ICD prevalence and severity taking advantage of the national prospective observational French NS‐Park cohort.^23^ Secondly, it aimed to compare the CSAI/ICD association on a longitudinal basis to the one of a matched group of PD patients under oral antiparkinsonian treatment. Finally, we sought to evaluate non‐motor symptoms, and disability milestones progression of patients submitted to CSAI treatment. We hypothesize that initiation of CSAI therapy, possibly through reduction or discontinuation of oral dopamine agonists, is associated with a decrease in ICD prevalence and severity in PD patients.

## Methods

### Study Design and Patients’ Assessment

This study utilized data from the French NS‐Park cohort, a national prospective observational registry encompassing 26 PD expert centers.^23^, ^24^

NS‐PARK is a national observational prospective cohort of patients followed in one of the 26 expert centers for PD in France. Inclusion in cohort is offered by neurologists, all experts in movement disorders, of participating centers, to all consecutive patients with a diagnosis of PD according to the MDS clinical diagnostic criteria.^25^ Demographic information, PD history, motor and non‐motor symptoms, and current antiparkinsonian treatment are collected at baseline (inclusion visit) and updated at each subsequent visit at the center. Motor symptoms, including motor fluctuations and dyskinesia and nonmotor signs, are assessed using scores derived from the MDS‐UPDRS part I–II and IV.^26^ PD severity is assessed using the Hoehn and Yahr scale (HY).^27^ Antiparkinsonian medications are recorded and classified according to pharmacological classes (amantadine, levodopa, DA, monoamine oxidase [MAO] or cathechol‐O‐methyl‐transferase [COMT] inhibitors), while a global levodopa equivalent daily dose (LEDD) is calculated according to international standard conversion formula^28^ and a LEDD for oral DA. Notably DA LEDD was assessed at each visit rather than as a cumulative dose.

Data are collected into a web‐based data capture system (RedCap) with regular data management and data extraction. The cohort was initiated in 2011 and declared to the French data agency (CNIL) by each center. In 2020, the protocol was updated to a formal national observational prospective cohort study, sponsored by Inserm, and the study received approval from an ethics committee (CPP IDF1, 16/06/2020, #IORG0009918) and recorded to clinicaltrials.gov (NCT04888364). All patients received written information about the study, and could express their opposition rights through the cohort website in accordance to EU General Protection Data Regulation rules (https://parkinsonnetwork/la-cohorte-ns-park) (https://parkinson.network/).

### Eligibility Criteria and Outcomes

For the present study, data collected from January 2016 up to October 2024 have been considered. Patients were eligible if they met MDS 2015 criteria for PD and had documented ICD status at visits before and after CSAI initiation.^29^ For the control group orally‐treated PD patients were selected based on matching for HY stage, ICD score, age and disease duration and visit period (see statistical methods for details on matching and propensity score analysis).

ICD were assessed by MDS‐UPDRS 1.6 derived score, on a 3‐unit grading (0 = no symptoms [0]; 1 = slight [1]; 2 to 4 = moderate/severe). The primary outcomes were ICD prevalence (ICD severity ≥1) and severity change comparing the visit before (baseline) and after CSAI initiation. Secondary outcomes were: (i) ICD progression comparing a CSAI‐treated population versus a matched group of orally‐treated PD patients; (ii) changes in motor fluctuations, dyskinesia, axial symptoms (dysarthria, postural instability, posture, swallowing problems), non‐motor symptoms (apathy, anxious mood, depressed mood, excessive day‐time sleepiness, orthostatic hypotension, constipation and urinary problems) and disability milestones (falls presence (ie, yes/no), hallucinations/psychosis and cognitive impairment) before and after CSAI initiation.

### Statistical Analysis

Baseline characteristics: Descriptive statistics of baseline demographic, clinical and therapeutic data were provided for continuous [median and interquartile range (IQR, 25^th^–75^th^ percentile)] and categorical (count and percentage) variables.

#### ICD Longitudinal Changes

For each patient, the closest visit before and the earliest visit surrounding CSAI initiation were selected (ie, two visits per patient). The proportion of patients on each level of ICD, together with the proportion with ICD ≥1, were compared before vs. after CSAI initiation. In addition, because this last comparison is not sensitive to patients who experienced an ICD decrease to a non‐zero landing score, the overall mean ICD scores were also compared before vs after CSAI initiation, which is in turn sensitive to (for instance) drops from ICD = 2 to ICD = 1. As a limitation of the real‐world nature of the data used in this study, the timing of visit/evaluation occurrence was significantly heterogeneous, with some patients having the “closest” visits several months before/after CSAI initiation (~50% within 2 years). Therefore, we performed sensitivity analyses and all comparisons before vs after CSAI initiation have been repeated with increasingly restrictive cohorts, whereby the allow elapsed time duration from evaluation to CSAI initiation (and vice versa) was progressively decreased (from a 60‐month to a 12‐month window).

#### Case–Control Analysis and Progression of Symptoms/Milestones

Paired/matched proportions were compared using McNemar's test. Paired/matched continuous scores were compared using the Wilcoxon signed‐rank test. In addition, given that most clinical sign and symptom scores are discrete in nature, these have also been compared using ordered logistic regression.

To evaluate the effect of CSAI on ICD scores over time, we performed a matching procedure to identify appropriate control patients who did not undergo CSAI therapy. The control group was selected based on strict matching criteria to ensure comparability with the CSAI group. Specifically, a maximum of five control patients for each CSAI patient were selected. These control patients must have had the exact same gender, HY stage, and ICD score, age and disease duration ±5 years, on a date within 31 days of the CSAI introduction (for each case) that also corresponded to an approximate sequential visit number at their respective centers (±2 visits).

Additionally, since this analysis was based on observational real‐world data, we used propensity score matching to assess the treatment effect while accounting for potential confounding factors. To ensure well‐defined treatment and control groups for comparison, we selected visits immediately before and after the initiation of CSAI for treated patients. Each treated patient was matched with five controls from the non‐CSAI‐treated group using a 1:5 nearest neighbor matching method. The propensity score was estimated using logistic regression, with treatment status as the dependent variable and a set of covariates as independent variables. These covariates included sex, age, disease duration, HY score, motor fluctuations, and dyskinesia, as well as the visit date. Notably, the exact visit date was also included in the propensity score calculation. The logistic regression model used a logit link function to calculate the propensity score, which represents the probability of receiving the treatment based on these covariates.

For the vast majority of the analyses, cases with missing key variables were excluded with no imputation being performed. Doing so resulted in minimal data loss given that individual variables were studied independently. However, in the propensity score matching analysis, missing data were imputed using a principal component analysis (PCA)‐based approach because not all patients had all available variables complete required for the matching procedure. This method allowed us to estimate missing values based on observed data structure, thereby preserving sample size and the integrity of the matching process.

Data were analyzed using R (v4.0.4) and Python.

## Results

### Baseline Characteristics

**Figure 1:**
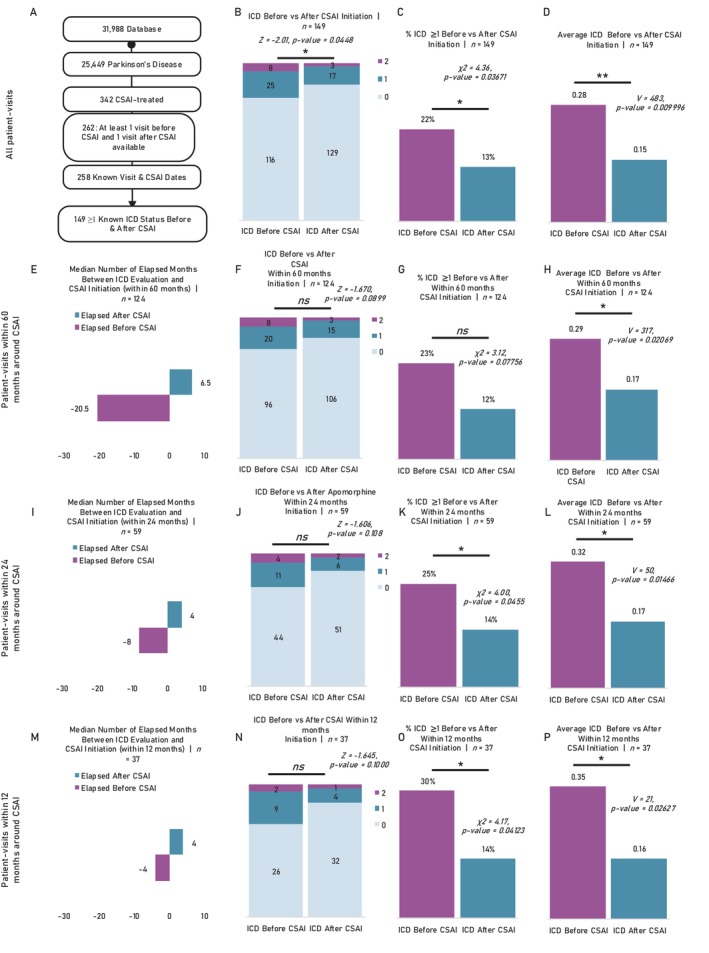
Impulse control disorder (ICD) longitudinal changes. (A) Patient funnel with enrollment criteria for ICD assessment before and after continuous subcutaneous apomorphine infusion (CSAI) introduction. Each patient must have had at least one visit before and one visit after CSAI introduction with known ICD status. For all subsequent analyses in this figure, only one visit is taken per patient before and after CSAI introduction (the ones closest to the event) so that all the analyses are paired. Figures (B), (C), and D) have no temporal domain restrictions. (B) Exact number of patient with ICD = 0,1, or 2 on the evaluations closest to CSAI introduction (before and after). Exact scores distribution before vs after CSAI initiation were compare using ordered logistic regression. (C) Percentage of patient with ICD ≥1 before and after CSAI introduction. Paired/matched proportions compared using the McNemar's test. (D) Average ICD before and after CSAI introduction. Paired/matched scores compared using the Wilcoxon signed‐rank test. Figures (E), (F), (G), and (H) have both visits pre‐ and post‐CSAI initiation restricted to a 60‐month window from the event. (E) Median number of month from ICD evaluation and CSAI initiation or from CSAI initiation and subsequent ICD evaluation (60‐month window restricted). (F) Exact number of patient with ICD = 0,1, or 2 on the evaluations closest to CSAI introduction (before and after, 60‐month window restricted). Exact scores distribution before vs after CSAI initiation were compared using ordered logistic regression. (G) Percentage of patient with ICD ≥1 before and after CSAI introduction. Paired/matched proportions compared using the McNemar's test (60‐month window restricted). (H) Average ICD before and after CSAI introduction. Paired/matched scores compared using the Wilcoxon signed‐rank test (60‐month window restricted). Figures (I), (J), (K), and (L) have both visits pre‐ and post‐CSAI initiation restricted to a 24‐month window from the event. (I) Median number of month from ICD evaluation and CSAI initiation or from apomorphine initiation and subsequent ICD evaluation (24‐month window restricted). (J) Exact number of patient with ICD = 0,1, or 2 on the evaluations closest to CSAI introduction (before and after, 24‐month window restricted). Exact scores distribution before vs after CSAI initiation were compared using ordered logistic regression. (K) Percentage of patient with ICD ≥1 before and after CSAI introduction. Paired/matched proportions compared using the McNemar's test (24‐month window restricted). (L) Average ICD before and after CSAI introduction. Paired/matched scores compared using the Wilcoxon signed‐rank test (24‐month window restricted). Figures (M), (N), (O), and (P) have both visits pre‐ and post‐CSAI initiation restricted to a 12‐month window from the event. (M) Median number of month from ICD evaluation and CSAI initiation or from CSAI initiation and subsequent ICD evaluation (12‐month window restricted). (N) Exact number of patient with ICD = 0, 1, or 2 on the evaluations closest to CSAI introduction (before and after, 12‐month window restricted). Exact scores distribution before vs after CSAI initiation were compared using ordered logistic regression. (O) Percentage of patient with ICD ≥1 before and after CSAI introduction. Paired/matched proportions compared using the McNemar's test (12‐month window restricted). (P) Average ICD before and after CSAI introduction. Paired/matched scores compared using the Wilcoxon signed‐rank test (12‐month window restricted). *ns* non‐significant, *P‐value<0.05, **P‐value<0.01, ***P‐value<0.001.

Over a total of 25.449 patients, 149 PD patients have been considered for our analysis based on inclusion criteria (see Fig. 1, panel A, for study flowchart) (59% male; mean age: 61.8 ± 10.6 years; mean disease duration: 8.1 ± 5.9 years). At inclusion, 40% of patients were receiving oral DAs, and the mean levodopa equivalent daily dose (LEDD) was 611 ± 481 mg and a mean LEDD for DA was 226 (±211) mg. Table 1 provides a detailed overview of baseline patients’ characteristics.

**TABLE 1:** Baseline clinical and demographic summary for continuous subcutaneous apomorphine infusion (CSAI)‐treated patients Cohort (n = 149) at visits immediately pre CSAI introduction before CSAI

Age: Mean (±SD) | Median [Q1‐Q3]	61.8 (±10.6) | 63 [55–70]
Sex (Male %)	59%
Disease Duration: Mean (±SD) | Median [Q1‐Q3]	8.1 (±5.9) | 7 [4–12]
H&Y (Med On): 1–2 | 3–5 | 2 NA(1%)	**1–2:** 73% | **3–5:** 27%
Impulse control disorders: 0 | 1 | ≥ 2 | [0NA(0%)]	**0:** 78% | **1:** 17% | ≥ **2:** 5%
Pain: 0 | 1 | ≥ 2 | [20NA(13%)]	**0:** 51% | **1:** 26% | ≥ **2:** 25%
Motor fluctuations: 0 | 1 | ≥ 2 | [4NA(3%)]	**0:** 22% | **1:** 49% | ≥ **2:** 29%
Dyskinesia: 0 | 1 | ≥ 2 | [4NA(3%)]	**0:** 42% | **1:** 41% | ≥ **2:** 17%
Somnolence: 0 | 1 | ≥ 2 | [5NA(3%)]	**0:** 59% | **1:** 31% | ≥ **2:** 10%
Orthostatic hypotension: 0 | 1 | ≥ 2 | [27NA(18%)]	**0:** 83% | **1:** 15% | ≥ **2:** 3%
Freezing of gait: 0 | 1 | ≥ 2 [5NA(3%)]	**0:** 68% | **1:** 19% | ≥ **2:** 12%
Dysarthria: 0 | 1 | ≥ 2 | [7NA(5%)]	**0:** 62% | **1:** 30% | ≥ **2:** 8%
Postural Instability: 0 | 1 | ≥ 2 | [7NA(5%)]	**0:** 65% | **1:** 26% | ≥ **2:** 9%
Postural Deformation: 0 | 1 | ≥ 2 | [13NA(9%)]	**0:** 75% | **1:** 18% | ≥ **2:** 6%
Hallucinations: 0 | 1 | ≥ 2 | [6NA(4%)]	**0:** 94% | **1:** 4% | ≥ **2:** 2%
Swallowing Problems: 0 | 1 | ≥ 2 | [8NA(5%)]	**0:** 89% | **1:** 10% | ≥ **2:** 1%
Falls: Yes | [5NA(3%)]	**Yes**: 19%
Digestive dysautonomia: 0 | 1 | ≥ 2 | [11NA(7%)]	**0:** 65% | **1:** 23% | ≥ **2:** 11%
Urinary dysautonomia: 0 | 1 | ≥ 2 | [10NA(7%)]	**0:** 71% | **1:** 24% | ≥ **2:** 5%
Apathy: 0 | 1 | ≥ 2 | [4NA(3%)]	**0:** 90% | **1:** 9% | ≥ **2:** 1%
Depression: 0 | 1 | ≥ 2 | [3NA(2%)]	**0:** 70% | **1:** 25% | ≥ **2:** 5%
Anxiety: 0 | 1 | ≥ 2 | [2NA(1%)]	**0:** 61% | **1:** 31% | ≥ **2:** 8%
LEDD (mg): Mean (±SD) | Median [Q1‐Q3]	611 (±481) | 450 [236–913]
DAAs: Yes (%)	40%
LEDD DAA (mg): Mean (±SD) | Median [Q1‐Q3]	226 (±211) | 206 [113–305]
**NA**: Not available	

NA: Not available.

### ICD Longitudinal Changes

At inclusion, 17% (n = 25) of the patients had slight ICDs, while 5% (n = 8) had mild to severe ICDs. Across the entire study population (n = 149), both the average ICD score and the proportion of patients with ICD ≥1 significantly decreased in the visit after CSAI introduction, independently on follow‐up delay (p = 0.003) (Fig. 1B–D). Ten patients (8%) transitioned from no ICD to ICD, including nine who moved from 0 to 1 and one from 0 to 2 (Fig. S1). Meanwhile, 23 patients (15%) shifted from ICD to no ICD, with 20 moving from 1 to 0 and three from 2 to 0. A total of 113 patients (75%) remained unchanged (106 at 0, five at 1, and two at 2), while three patients (2%) experienced a reduction (from 2 to 1). Similarly, this pattern held true across progressively more restrictive cohorts, particularly in evaluations conducted closer to the pump introduction event (Fig. 1E–P). If 20%–30% of patients presented an ICD ≥1 on the evaluation immediately before CSAI introduction, this prevalence decreased to 10%–15% on the visit after CSAI initiation.

**Figure 2:**
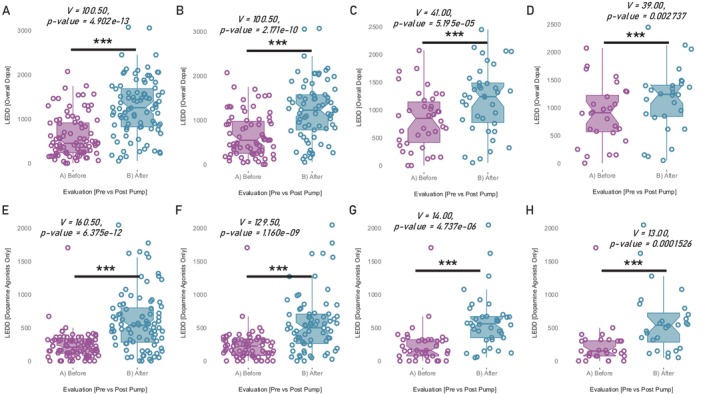
Levodopa equivalent daily dose (LEDD) longitudinal changes. (A) Overall LEDD before vs after continuous subcutaneous apomorphine infusion (CSAI) introduction (entire cohort). (B) Overall LEDD before vs after CSAI introduction (patients with known non‐zero LEDD within the flaking 60 months). (C) Overall LEDD before vs after CSAI introduction (patients with known non‐zero LEDD within the flaking 24 months). (D) Overall LEDD before vs after CSAI introduction (patients with known non‐zero LEDD within the flaking 12 months). (E) Dopaminergic Agonist (DA) LEDD before vs after CSAI introduction (entire cohort). F) DA LEDD before vs after CSAI introduction (patients with known non‐zero LEDD within the flaking 60 months). (G) DA LEDD before vs after CSAI introduction (patients with known non‐zero LEDD within the flaking 24 months). (H) DA LEDD before vs after CSAI introduction (patients with known non‐zero LEDD within the flaking 12 months). *ns* non‐significant, *P‐value<0.05, **P‐value<0.01, ***P‐value<0.001.

**Figure 3:**
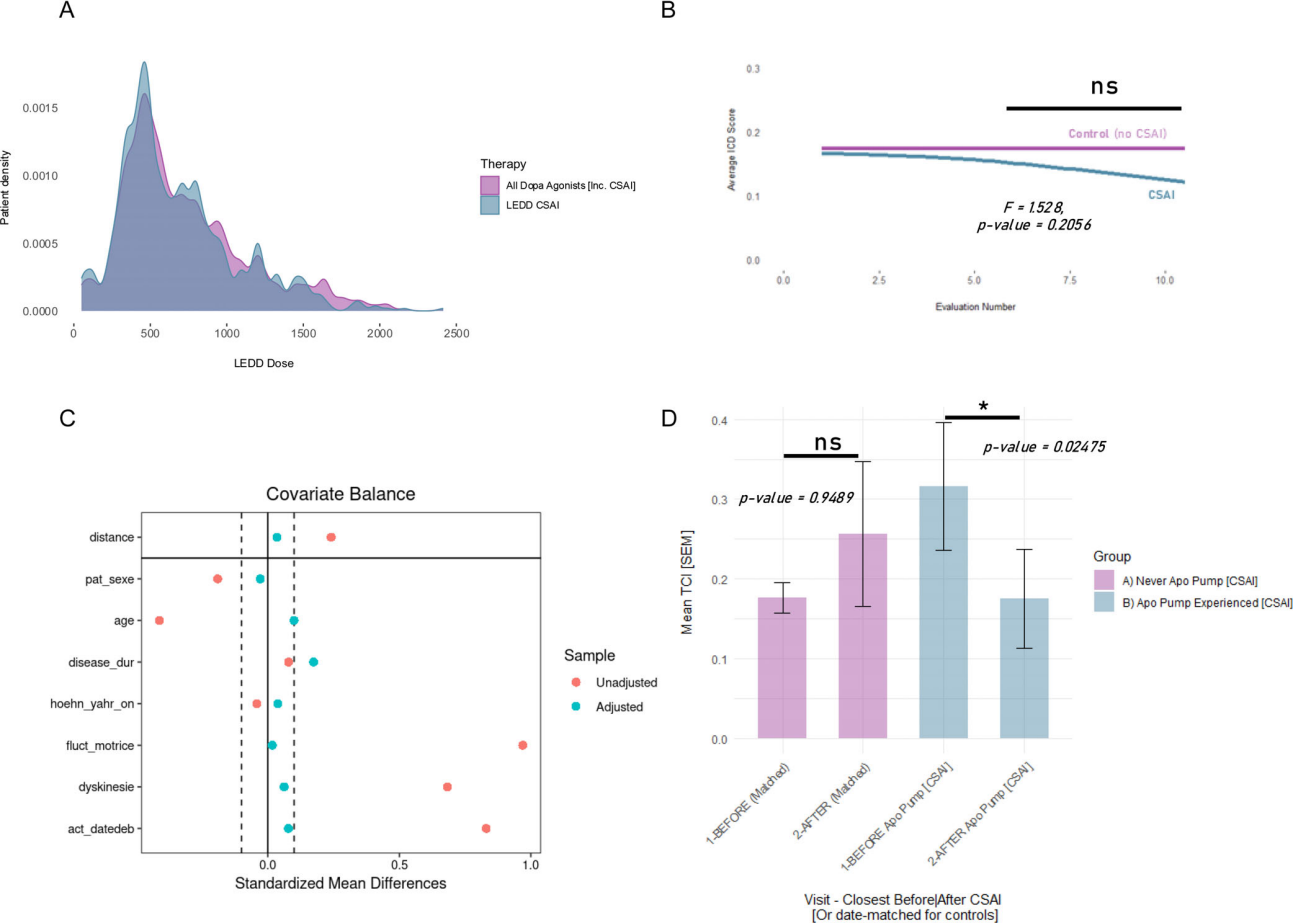
Case control analysis. (A) Continuous subcutaneous apomorphine infusion (CSAI) levodopa equivalent daily dose (LEDD) and total dopamine agonists (DA) (including CSAI) LEDD distribution for all available patient‐visits with known/recorded doses (n = 677 patient‐visits post‐CSAI initiation). (B) Locally weighted regression fitting of Impulse control disorder (ICD) scores for patients who have been put on a CSAI (at some point during their trajectory) and respective control patients (1:5) over the sequential visit/evaluation number. No temporal restrictions applied. The control group was selected based on strict matching criteria to ensure comparability with the pump group. Specifically, a maximum of five control patients for each CSAI patient were selected. These control patients must have had the exact same gender, ON Hoehn & Yahr, and ICD score, age and disease duration ±5 years, on a date within 31 days of the CSAI introduction (for each case) that also corresponded to the same sequential patient visit at the center (±5 visits). While the trajectories seem to diverge, no statistically significant interactions was observed between the “group” and “time” variables upon linear effects modeling. (C) Love plot showing the balance of covariates before and after propensity score matching. The plot visualizes standardized mean differences for each covariate, where the matching procedure has improved the covariate balance between the treatment and control groups. (D) Bar plot of mean ICD scores with standard error comparing the treatment (CSAI) and control (never CSAI) groups. The bars represent the mean ICD score at each visit, with error bars indicating the standard error of the mean. To assess the treatment effect while accounting for potential confounding factors using propensity score matching, CSAI‐treated patients had their visits immediately before and after the initiation of treatment selected, ensuring that the treatment and control groups were well‐defined for comparison. For each of the treated patients, we matched five controls from the non‐treated group using a 1:5 nearest neighbor matching method. The propensity score was estimated using logistic regression, with treatment status as the dependent variable and a set of covariates as independent variables. These covariates included sex, age, disease duration, Hoehn & Yahr score, motor fluctuations, and dyskinesia, as well as the visit date. Notably, the exact visit date was also included in the propensity score calculation. The logistic regression model used a logit link function to calculate the propensity score, which represents the probability of receiving the treatment based on these covariates. Paired/matched scores compared using the Wilcoxon signed‐rank test (rank‐sum test in the case of independent samples—controls). *ns* non‐significant, *P‐value<0.05.

The observed reductions in ICD occurred despite a significant increase in the total dopaminergic agonist LEDD (Table S1 and Fig. 2). Here, when looking at the total dopaminergic agonist LEDD at the visits after CSAI introduction, one observes the CSAI to account for the total dopaminergic agonist LEDD in its almost entirety (with an average residual difference of 42.3 ± 95.8 mg, Fig. 3A). This residual difference of dopaminergic agonists other than the CSAI is, not surprisingly, dramatically lower (<1/5th) than that observed at the pre CSAI visits (regardless of the cohorts or time constraints enforced, ie, 226 ± 211 mg on Table S1).

### Case Control Analysis

When comparing patients who have started CSAI with matched controls, the control patient group had a stable ICD score over the different evaluations, conversely to the CSAI cohort, in which an ICD decrease was observed, though the two trajectories were not statistically different (Fig. 3B). Notably, considering all visits, the mean DA LEDD among matched controls was 169 ± 130 mg, whereas it reached 362 ± 492 mg in the CSAI‐treated population. The same hold true when applying a propensity score matching (Fig. 3C,D) showing a longitudinal decrement of ICD in the CSAI‐treated group (on the right) and an inverse trend in the orally treated group (on the left).

### Progression of Motor, Non‐motor Symptoms and Disability Milestones

Comparisons of visits separated by longer intervals (60 months of follow‐up) revealed an overall mild, though significant, increase in motor complications compared to pre‐CSAI initiation (1.13 ± 0.83 vs. 1.43 ± 0.90 for motor fluctuations and 0.77 ± 0.78 vs. 1.17 ± 0.94 for dyskinesia). In contrast, when focusing on pre‐ and post‐CSAI visits much closer to it (within 24 or 12 months), no global significant differences were observed for motor complications from the pre‐ to the post‐CSAI evaluations, but a reduction of the most troublesome (score ≥3) motor fluctuations (from 14% to 2% for 24 months and from 17% to 6% for 12 months) (Table S2).

Likewise, a statistically significant mild worsening was found at 60 months but not 12–24 months visits for: dysarthria, freezing of gait, postural instability, posture, swallowing problems, orthostatic hypotension and urinary disorders, apathy, depression, anxiety, hallucinations, excessive daytime‐somnolence and cognitive disturbance (Table S2). Falls rate does not show significant changes over the whole follow‐up if compared to pre‐CSAI, showing a baseline frequency around 20% and occurring in about 30% of the patients at the latest follow‐up. Of note, no significant differences were found at the levels of pain intensity and/or overall pain prevalence at 60, 24 or 12 months.

## Discussion

Our cohort study finds a significant reduction in both ICD scores and the proportion of advanced PD patients with ICD ≥1 following the initiation of CSAI therapy over a follow‐up ranging from 12 to 60 months. Of note, the majority of baseline ICDs were slight with a minor proportion of patients (5%) presenting mild to severe ICDs, which is consistent with the expected distribution based on the criteria for CSAI treatment selection. ICD attenuation was observed consistently across the entire cohort and within subgroups with increasingly restrictive temporal windows, highlighting the robustness of its effects. These improvements occurred despite an increase in the total DA dose, suggesting that CSAI may mitigate mild behavioral complications typically associated with such therapies without increasing apathy and mood disorders. Notably, our longitudinal case–control analysis confirms that CSAI treatment is associated with a trend in ICD reduction despite a higher DA LEDD, if compared to a matched orally treated population.

The significant reduction in ICD prevalence and severity following CSAI initiation in our cohort corroborates findings from previous studies which reported behavioral symptom stabilization or improvement.^9^, ^10^, ^16^, ^30^ These results are particularly compelling given the increased dopaminergic load inherent to CSAI treatment and possibly associated to the concomitant tapering of oral DA dose, which reduces mesolimbic sensitization.^31^ For instance, Todorova et al^11^ and Barbosa et al^12^ demonstrated similar behavioral benefits, underscoring CSAI potential to improve behavioral complications without exacerbating motor dysfunction. This contrasts sharply with the effects of oral DA such as pramipexole and ropinirole that have been showed to be strongly associated with high ICD prevalence, with rates reported between 17% and 51.5% depending on therapy duration, dose and cumulative dose.^6^, ^32^, ^33^ The pharmacokinetic and pharmacodynamic properties of these agents likely explain this disparity. Pramipexole's high bioavailability and potent affinity for mesolimbic D3 receptors predispose patients to ICD development irrespective of serum concentrations.^32^ In contrast, ICD risk among ropinirole users seems closely linked to serum concentration peaks, highlighting the importance of therapeutic drug monitoring.^3^, ^32^

Our study also targets a specific PD population and underscores the necessity of rigorous clinical criteria for CSAI initiation. Typically, patients selected for CSAI are in an advanced PD stage, where ICD risk can be mitigated through discontinuation of oral DA and implementing thorough strict patients monitoring. This is not the case for early and young PD patients, less likely to receive a close monitoring and less warned on ICD risk. This is consistent with findings by Drapier et al,^16^ who observed quality‐of‐life improvements alongside reductions in behavioral and non‐motor complications with CSAI use. Additionally, Barbosa et al^13^ reported enhanced treatment adherence and further reductions in ICD prevalence among CSAI users. Nevertheless, rare instances of new‐onset ICD following CSAI initiation have been documented, necessitating therapy discontinuation in some cases.^9^, ^13^, ^15^ These findings underscore the importance of recognizing individual predispositions, such as genetic polymorphisms in dopamine receptor pathways, as potential mediators of ICD risk and to continue a strict monitoring of those symptoms.^15^, ^32^

The distinct pharmacokinetic and pharmacodynamic properties of subcutaneous CSAI versus oral DAs likely underlie the observed differences in ICD risk, with CSAI offering more stable dopaminergic stimulation. Oral DA, exert strong selectivity for D2 and D3 receptors in the mesolimbic pathway—a circuit central to reward processing and impulsivity.^8^, ^32^ Chronic overstimulation of D3 receptors is closely linked to maladaptive reward behaviors, significantly increasing ICD risk.^21^, ^32^ In contrast, CSAI's continuous infusion delivers stable dopaminergic stimulation, avoiding the pulsatile receptor activation that characterizes oral DA.^1^, ^18^ This steady activation likely prevents mesolimbic sensitization and associated maladaptive plasticity, key processes in ICD pathogenesis. Additionally, CSAI preferentially targets presynaptic dopamine autoreceptors, providing a regulatory effect on dopaminergic transmission that further reduces ICD risk.^11^, ^17^

The presented results have potential implications for clinical decision‐making in PD management. Given that ICD symptoms appeared to decrease following CSAI initiation, possibly due to the reduction or discontinuation of oral DA and the continuous dopaminergic stimulation allowed by the pump, CSAI may be a possible treatment option for patients at high risk of ICDs, (eg, young, male and personal and/or familial history of mood disorders and addiction^34^), particularly those with oral dopamine agonists. However, it is important to consider that increased medical oversight and enhanced patient adherence associated with pump therapy could also influence these outcomes. Additionally, the initiation of CSAI remains a treatment option that should be discussed with patients. It may be associated with adverse events, such as cutaneous reactions, which are not present with oral therapies and may be perceived by some patients as more invasive.

Non‐motor symptoms classically associated with CSAI, such as excessive daytime sleepiness, orthostatic hypotension, and hallucinations, show only a slight increase during longer follow‐up periods. This likely reflects that our CSAI population represents a subset of advanced PD patients who tolerate the treatment well, with disease milestones and non‐motor symptoms progression emerging only over time, likely due to natural disease progression. Interestingly, falls frequency does not increase, in line with recent systematic analyses that suggest a potential reduction of falls associated with continuous subcutaneous infusion, particularly when compared to deep brain stimulation.^35^

The main limitation of this study lies in its observational design, which restricts causal inference. Additionally, we did not collect reasons for CSAI drop‐out, inducing the possible bias of considering a PD population who tolerated this treatment well and excluding patients who may have presented major non‐motor symptoms complications. Moreover, this study relied on individual MDS‐UPDRS sub‐items to assess impulse control disorders, which is suboptimal compared to the use of validated ICD‐specific scales such as the QUIP or Ardouin Scale. However, due to the retrospective and multicenter nature of the dataset,—applied to a large population of patients at each follow‐up visit—, standardized assessments were not consistently available neither feasible. As a result a MDS‐UPDRS item was adopted as the most feasible proxy for capturing clinically relevant impulsive‐compulsive behaviors. These limitations notwithstanding, we present data of a large national cohort collected in PD tertiary expert centers, well in line with previous smaller studies.

In conclusion, our findings may contribute to a growing consensus on the potential of CSAI to attenuate ICD, while targeting advanced PD patients. These improvements were consistent across various subgroups and observed despite an overall increase in dopaminergic load. This highlights CSAI's ability to mitigate behavioral complications associated with oral DA without negatively impacting mood. While this observation is clinically relevant, it should be interpreted with caution given the study's observational design and the limitations inherent to using MDS‐UPDRS sub‐items for ICD assessment. Further prospective, controlled studies using standardized ICD scales are needed to confirm these findings and clarify the underlying mechanisms. Nonetheless, these findings may inform treatment decisions by suggesting that CSAI could be a particularly suitable option for PD patients with existing or emerging ICD related to oral dopamine agonist use—especially when further dopaminergic therapy is needed but ICD risk remains a concern.

## Author Roles

(1) Research project: A. Conception, B. Organization, C. Execution; (2) Statistical Analysis: A. Design, B. Execution, C. Review and Critique; (3) Manuscript Preparation: A. Writing of the first draft, B. Review and Critique;

C.D.: 1A, 1B, 1C, 2C, 3A, 3B.

P.A.D.B.: 1A, 1B, 2A, 2B, 2C, 3B.

A.L.: 2C, 3B.

C.B.C.: 1A, 1B, 3B.

I.B.: 1B, 3B.

C.G.: 1B, 3B.

A.D.M.: 1B, 3B.

G.C.: 1B, 3B.

P.R.: 1B, 3B.

L.D.: 1B, 3B.

C.T.: 1B, 3B.

S.P.: 1B, 2C, 3B.

J.L.H.: 1B, 3B.

A.S.F.: 1B, 3B.

F.O.M.: 1B, 3B.

R.P.B.: 1B, 3B.

N.B.: 1B, 3B.

J.C.C.: 1A, 1B, 1C, 2C, 3B.

O.R.: 1A, 1B, 1C, 2C, 3B.

M.F.: 1A, 1B, 1C, 2C, 3A, 3B.

## Disclosures

**Ethical Compliance Statement:** According to the French ethic and regulatory law (public health code) retrospective studies based on the exploitation of usual care data should be submitted to an ethic committee but they have to be declared or covered by reference methodology of the French National Commission for Informatics and Liberties (CNIL). The authors confirm that the approval of an institutional review board was not required for this work. All patients received written information about the study, and could express their opposition rights through the cohort website in accordance to EU General Protection Data Regulation rules (https://parkinsonnetwork/la-cohorte-ns-park) (https://parkinson.network/). A collection and computer processing of personal and medical date was implemented to analyze the results of the research. Toulouse University Hospital signed a commitment of compliance to the reference methodology MR‐004 of the French National Commission for Informatics and Liberties (CNIL). After evaluation and validation by the data protection officer and according to the General Data Protection Regulation*, this study completing all the criteria, it is registered in the register of data study of the Toulouse University Hospital (number's register: RnIPH 2023–39) and covered by the MR‐004 (CNIL number: 2206723 v 0). We confirm that we have read the Journal's position on issues involved in ethical publication and affirm that this work is consistent with those guidelines.

**Funding Sources and Conflict of Interest:** No specific funding was received for this work. The authors declare that there are no conflicts of interest relevant to this work.

**Financial Disclosures for the Previous 12 Months:** CD, PADB, AL, IB, CG, ADdM, GC, PR, SP, J‐LH, ASF, RPB, NB, report no disclosures. MF received Honoraria to speak from AbbVie, ORKYN, and BIAL, consultancies from BIAL and LVL Medical; Grant from France Parkinson, HORIZON 2022 French Ministry of Health and MSA Coalition. CB‐C has received research grant from Association France Parkinson, and fees for lectures and consultancies from Aguettant, Orkyn, NHC, Zambon and AbbVie. LD served on the Scientific Advisory Board for Abbvie and has received honoraria from pharmaceutical companies for consultancy and lectures including Abbvie, Novartis, Aguettant, Orkyn. FO‐M has received honoraria for serving as an advisory board member from Abbvie, Medtronic, Orphalan, Aguettant and Orkyn, and for consultancy activities from Aguettant, Abbvie, Orphalan, Ellivie, Homeperf and Orkyn. OR has acted as a scientific advisor for drug companies developing antiparkinsonian medications (Abbott, Abbvie, Acorda, Adamas, BIAL, Biogen, Boehringer‐Ingelheim, Cynapsus, GSK, Impax, Merck, Osmotica, Oxford‐Biomedica, Lundbeck, Novartis, Prexton, Servier, Sunovion, TEVA, UCB, Zambon). CT reports no financial disclosure; congress travel grant from Orkyn, Abbvie, homeperf, ADELIA, ASDIA, Ipsen.

## Supporting information


**Figure S1.** Evolution of Impulse control disorder (ICD) prevalence and severity over time. (A) Exact number of patients per ICD score/severity before vs after CSAI (all patient visits). (B) Exact number of patients per ICD score/severity before vs after CSAI (patient visits within 60 months of CSAI initiation). (C) Exact number of patients per ICD score/severity before vs after CSAI (patient visits within 24 months of CSAI initiation). (D) Exact number of patients per ICD score/severity before vs after CSAI (patient visits within 12 months of CSAI initiation).

**TABLE S1.** Summary dopaminergic Agonist LEDD scores before vs after CSAI initiation.

**TABLE S2.** Motor, axial, non‐motor and disability milestones scores before and after CSAI initiation.

## Data Availability

The data that support the findings of this study are available on request from the corresponding author. The data are not publicly available due to privacy or ethical restrictions.

## APPENDIX A

**Executive Board:**

Jean‐Christophe Corvol (Coordinator, *Paris—Pitié Salpêtrière*), Olivier Rascol (*CHU Toulouse*), Margherita Fabbri (*CHU Toulouse*), Wassilios Meissner (*CHU Bordeaux*), Stéphane Thobois *(CHU Lyon)*, Luc Defebvre *(CHU Lille)*, Helene Esperou (*INSERM*), Florence Tubach (*Cephépi, AP‐HP*), Yann De Rycke (*Cephépi, AP‐HP*).

**Management group:**

Coordination (*ICM, Paris*): Stephanie CARVALHO, Yajiththa RAJASEGARAM; Sponsor (*INSERM*): Layidé Roufai, Dalila Ouazib, Sandra Zarrad; NS‐PARK network (*CHU Toulouse*): Fatma Khelifi, Nacim MIRI.

**Datacore:**

Coordination (*Cephépi, AP‐HP*): Florence Tubach, Yann De Rycke; Statistics (*Cephépi, AP‐HP*): Nathalie Bertille; (*CHU Toulouse*): Vanessa Rousseau, Samuel Tessier, Margherita Fabbri; Data management (*Cephépi, AP‐HP*): David Tavel, Dounya Metdaoui; (*ICM, Paris*): Avigaelle Abitbol, Mathias Antunes.

**Genetics team**.

*(ICM, Paris)*: Alexis Brice, Suzanne Lesage, Christelle Tessson, Fanny Casse, Mélanie Ferrien, Guillaume Cogan, Lisa Welment.

**Principal Investigators**.

(CHU Alphabetical Order): *Aix‐en‐Provence*: Silvia DI LEGGE; *Amiens*: Melissa TIR; *Avicenne Hospital*: Bertrand DEGOS; *Besançon*: Thierry Moulin; *Bordeaux*: Wassilios Meissner; *Caen*: Claire Thiriez; *Clermont‐Ferrand*: Ana MARQUES; *Créteil*—*Henri Mondor*: Philippe REMY; *Dijon*: Gwendoline DUPONT; *Grenoble*: Elena MORO; *Lille*: Luc DEFEBVRE; *Limoges*: Jean‐Luc HOUETO; *Lyon*: Stéphane Thobois; *Marseille*: Jean‐Philippe AZULAY; *Montpellier*: Christian Geny; *Nancy*: Solène Frismand; *Nantes*: Anne‐Gaëlle Corbille; *Nice*: Caroline Giordana; *Nîmes*: Giovanni Castelnovo; Paris—*Pitié*—*Salpêtrière*: Jean‐Christophe CORVOL; *Poitiers*: Isabelle Benatru; *Reims*: Anne DOE DE MAINDREVILLE; *Rennes*: Sophie Drapier; *Rouen*: David Maltete; *Strasbourg*: Christine Tranchant; *Toulouse*: Olivier Rascol.

**Co‐investigators:**

*Amiens*: Mickael Aubignat; *Avicenne Hospital*: Marie Mongin, Arnaud LAPOSTOLLE; *Besançon*: Matthieu Bereau, Gautier CLEMENT; *Bordeaux*: Alexandra Foubert‐Samier, Brice Laurens, Sylvain Vergnet, Thomas Boraud, David Bendetowicz, Jade Sarrabere, Dominique Guehl, Pierre Burbaud, Edouard COURTIN; *Caen*: Paul‐Alexandre Pfeiffer, Gilles Defer; *Clermont‐Ferrand*: Bérengère Debilly, Philippe Derost, Charlotte BEAL; *Créteil*—*Henri Mondor*: Hayet SALHI, Alice Dormeuil, Aimée Petit, Alban Gravier; *Dijon*: Vincent Schneider, Lucie Garnier; *Lille*: Nicolas Carriere; *Limoges*: Olivier Colin, Philippe Couratier; *Lyon*: Chloé Laurencin, Stephane Prange, Paul Jaulent, Bruno Plus, Helene Gervais‐Bernard; *Marseille*: Alexandre Eusebio, Frédérique Fluchere, Stephan Grimaldi, Valentin Mira, Tatiana Witjas; *Montpellier*: Mahmoud Charif, Ophelie Forster, Alix Durand, Pauline Prin; *Nancy*: Lucie Hopes, Amory Jardel, Salome Puisieux, Guillemette Clement; *Nantes*: Philippe Damier, Tiphaine Rouaud, Pascal Derkinderen, Arthur Lionnet, Adrien De Guilhem De Lataillade; *Nice*: Cosmin Alecu, Charlotte Heraud; *Nîmes*: Marie De Verdal; Paris—*Pitié*—*Salpêtrière*: Graziella Mangone, Sara Sambin, Aymeric Lanore, Thomas Courtin, Louise‐Laure Mariani, Fouad Khoury, Poornima Menon, Florence Cormier‐Dequaire, Emmanuel Flamand‐Roze, David Grabli, Elodie Hainque, Marie Vidhaillet, Aurélie Meneret, Cécile Delorme, Cendrine Foucard, Florian Von Raison, Alexis Elbaz, Andreas Hartmann, Vincent Leclercq, Theodore Soulier, Daniel Torres, Giulia Coarelli, Giorgia Querin, Fabien Hauw, Margaux Dunoyer; *Poitiers*: Solène Ansquer; *Rennes*: Frederique Leh, Marion Leclercq, Simon Lamy; *Reims*: Guillaume Carey; *Rouen*: Guillaume Costentin, Clemence Hardy; *Strasbourg*: Mathieu Anheim, Ouhaid Lagha Boukbiza, Thomas Wirth, Jimmy Voirin, Marie Des Neiges Santin, Thomas Bogdan; *Toulouse*: Margherita Fabbri, Fabienne Ory‐Magne, Christine Brefel Courbon, Clemence Leung, Hélène Catala, Gabrielle Sill, Raquel Pinheiro Barbosa.

**Data Collection Contributors**.

(Project Managers, Clinical Research Associate, Study Nurses, Secretary, Neuropsychologist): *Aix‐en‐Provence*: Lucy Famer; *Avicenne Hospital*: Lucie Braccagni, Hiba Sifaoui, Juliette Palisson, Kenza Benrahmoune Idrissi; *Amiens*: Astrid Causel, Lydie Romeo, Constance Bissessur; *Besançon*: Audace Cure‐Martin, Charline Compagne; *Bordeaux*: Sandrine Dupouy, Sandrine Villars, Wei‐Ho Lai; *Caen*: Rachida Bari, Damien Chevanne; *Clermont‐Ferrand*: Elodie Durand, Isabelle Rieu, Stephane Bernard, Corinne Garsault, Nathalie Meunier; *Créteil*—*Henri Mondor*: Alexia Cresson, Marine Sgard, Marie Dreano, Justine Montillot, Renaud Massart; Dijon: Pascale Grebent; *Grenoble*: Pierre Pelissier; *Lille*: Valérie Santraine; *Limoges*: Thomas Gaudin, Pierre Boutet, Cécile Thuilier, Coralie Chalot, Céline Prevost, Hélène Videaud, Justine Picut, Christian Tarrade; *Lyon*: Catherine Caire, Hélène Merle, Elise Metereau, Mathilde Millot, Chloe Bernardi, Emilie Favre, Adelaide Jaulent; *Marseille*: Laura Mundler, Blandine Dufresne, Eve Benchetrit; *Montpellier*: Valérie Driss, Alexia Arifi, Maura Rodrigues; *Nancy*: Lili Le Monnier, Nathalie Dumont, Virginie Bablon; *Nantes*: Régis Frenais, Caroline Herve, Christelle Guimber; *Nice*: Vanessa Ferrier, Elodie David, Christina Faroul; Nîmes: Leslie Fra, Elsa Foucaran, Fatima‐Ezzahra Ennaji; *Paris‐Pitié‐Salpêtrière*: Jeremy Bonetto, Ryad Ladghem‐Chikouche, Mickael Le, Sophie Liot, Sonia Messar, Hamza Salah, Amelie Bernardo, Naoual Serari; *Poitiers*: Emilie Rabois, Carole David, Zoe Fournier; *Reims*: Margaux Bonnaire‐Verdier, Elise Chevaillier; *Rennes*: Françoise Kestens, Rozenn Gourhan, Sandra Lopez‐Alfaro, Jean‐François Houvenaghel, Mélanie Alexandre, Christine Bourdonnais; *Rouen*: Linda Vernon, Ahmed Boumediene; *Strasbourg*: Hugo Rummel, Céline Julie, Nadine Longato, Célie Phillipps, Anne Claire Andries‐Ros; *Toulouse*: Stéphanie Bras, Estelle Harroch, Claudia Gillet, Yoan Herades, Eva Camgrand.
