# Supplementary material for: Subcutaneous Apomorphine Infusion Initiation Is Associated with Impulse Control Disorder Attenuation in Advanced Parkinson's Disease Patients: Insights from the French NS‐Park Cohort
Source: Mov Disord Clin Pract. 2025 Jul 17;13(1):142–53. doi: 10.1002/mdc3.70240 (PMC12839497; doi:10.1002/mdc3.70240)

Supplementary Figure S1 - Evolution of Impulse control disorder (ICD) prevalence and severity over time.

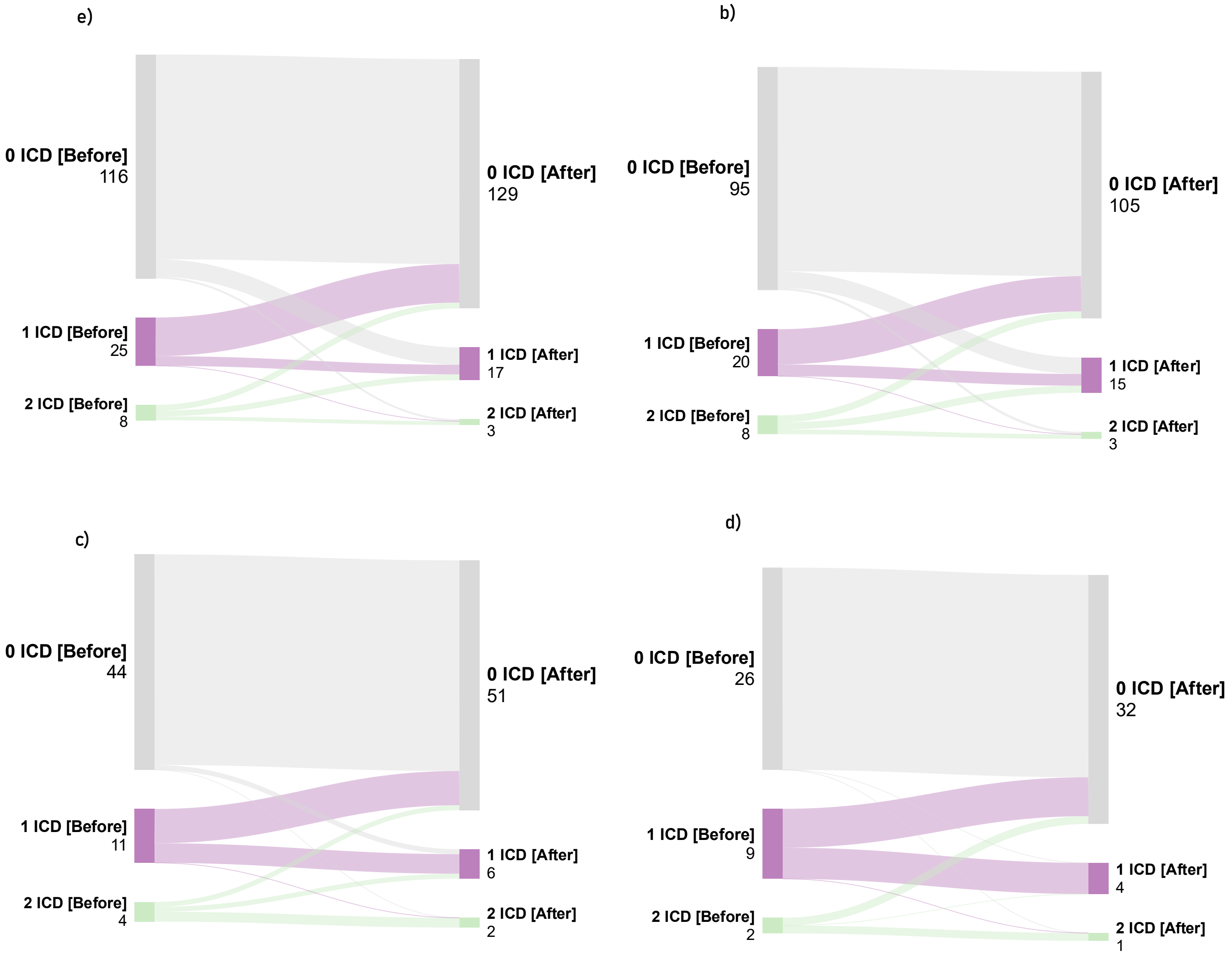

Supplement: Supplementary file 1 — Figure S1. Evolution of Impulse control disorder (ICD) prevalence and severity over time. (A) Exact number of patients per ICD score/severity before vs after CSAI (all patient visits). (B) Exact number of patients per ICD score/severity before vs after CSAI (patient visits within 60 months of CSAI initiation). (C) Exact number of patients per ICD score/severity before vs after CSAI (patient visits within 24 months of CSAI initiation). (D) Exact number of patients per ICD score/severity before vs after CSAI (patient visits within 12 months of CSAI initiation). [file MDC3-13-142-s002.pdf]
