# Supplementary material for: Subcutaneous Apomorphine Infusion Initiation Is Associated with Impulse Control Disorder Attenuation in Advanced Parkinson's Disease Patients: Insights from the French NS‐Park Cohort
Source: Mov Disord Clin Pract. 2025 Jul 17;13(1):142–53. doi: 10.1002/mdc3.70240 (PMC12839497; doi:10.1002/mdc3.70240)
Supplement: Supplementary file 2 — TABLE S1. Summary dopaminergic Agonist LEDD scores before vs after CSAI initiation. [file MDC3-13-142-s003.pdf]

Supplementary Table S1 - Summary Dopaminergic Agonist LEDD Scores Before vs After CSAI Initiation

Dopaminergic Agonist LEDD Scores [Mean ± SD | Median (Q1-Q3)]			
(Sub)population	Before CSAI	After CSAI	*p-value* ▲
Overall 88 Patients With LEDD Evaluation Before & After CSAI Initiation	226 ± 211<br>206 [113-305]	597 ± 447<br>538 [282-801]	*** <0.001
73 Patients With LEDD Evaluation Before & After CSAI Initiation within 60 months	231 ± 227<br>224 [80-320]	564 ± 435<br>500 [263-706]	*** < 0.001
38 Patients With LEDD Evaluation Before & After CSAI Initiation within 24 months	239 ± 294<br>160 [74-320]	593 ± 409<br>560 [351-671]	*** < 0.001
28 Patients With LEDD Evaluation Before & After CSAI Initiation within 12 months	235 ± 321<br>150 [74-305]	590 ± 471<br>538 [284-724]	*** < 0.001
**Overall Total LEDD Scores [Mean ± SD | Median (Q1-Q3)]**			
(Sub)population	Before CSAI	After CSAI	*p-value* ▲
Overall 88 Patients With LEDD Evaluation Before & After CSAI Initiation	611 ± 481<br>450 [236-913]	1264 ± 667<br>1257 [822-1686]	*** < 0.001
73 Patients With LEDD Evaluation Before & After Apomorphine CSAI within 60 months	662 ± 494<br>545 [250-974]	1219 ± 672<br>1227 [780-1574]	*** < 0.001
38 Patients With LEDD Evaluation Before & After Apomorphine CSAI within 24 months	822 ± 516<br>850 [415-1147]	1179 ± 608<br>1242 [776-1489]	*** < 0.001
28 Patients With LEDD Evaluation Before & After Apomorphine CSAI within 12 months	894 ± 532<br>910 [568-1223]	1147 ± 588<br>1242 [844-1406]	*** 0.002

▲ *Paired continuous scores compared using the Wilcoxon signed-rank test, respectively.* *NA*: Not applicable.
