# Supplementary material for: Subcutaneous Apomorphine Infusion Initiation Is Associated with Impulse Control Disorder Attenuation in Advanced Parkinson's Disease Patients: Insights from the French NS‐Park Cohort
Source: Mov Disord Clin Pract. 2025 Jul 17;13(1):142–53. doi: 10.1002/mdc3.70240 (PMC12839497; doi:10.1002/mdc3.70240)
Supplement: Supplementary file 3 — TABLE S2. Motor, axial, non‐motor and disability milestones scores before and after CSAI initiation. [file MDC3-13-142-s001.pdf]

Supplementary Table S2A- Summary Motor Complication & Axial Scores Before vs After CSAI Initiation

Motor Complication: Motor Fluctuations
[Mean ± SD | Breakdown (%)]

(Sub)population	Before CSAI	After CSAI	*p-value* ▲
Overall 145 Patients With Motor Fluctuations Evaluation Before & After CSAI Initiation	1.13 ± 0.83<br>0: 22% | 1: 49% | 2: 23% | 3: 6%	1.43 ± .90<br>0: 14% | 1: 42% | 2: 32% | 3: 10% | 4: 1%	** 0.0023<br>** 0.0038
120 Patients With Motor Fluctuations Evaluation Before & After CSAI Initiation within 60 months	1.23 ± 0.85<br>0: 19% | 1: 46% | 2: 28% | 3: 8%	1.47 ± 0.90<br>0: 13% | 1: 42% | 2: 34% | 3: 10% | 4: 2%	* 0.0312<br>* 0.0480
57 Patients With Motor Fluctuations Evaluation Before & After CSAI Initiation within 24 months	1.56 ± 0.82<br>0: 7% | 1: 44% | 2: 35% | 3: 14%	1.42 ± 0.82<br>0: 11% | 1: 46% | 2: 37% | 3: 5% | 4: 2%	0.2899<br>0.3630
36 Patients With Motor Fluctuations Evaluation Before & After CSAI Initiation within 12 months	1.61 ± 0.84<br>0: 6% | 1: 44% | 2: 33% | 3: 17%	1.31 ± 0.75<br>0: 11% | 1: 53% | 2: 31% | 3: 6%	0.0671<br>00.129

Motor Complication: Dyskinesias
[Mean ± SD | Breakdown (%)]

(Sub)population	Before CSAI	After CSAI	*p-value* ▲
Overall 145 Patients With Dyskinesias Evaluation Before & After CSAI Initiation	0.77 ± 0.78<br>0: 42% | 1: 41% | 2: 15% | 3: 2%	1.17 ± 0.94<br>0: 26% | 1: 43% | 2: 22% | 3: 9% | 4: 1%	*** <0.0001<br>*** 0.0003
120 Patients With Dyskinesias Evaluation Before & After CSAI Initiation within 60 months	0.83 ± 0.82<br>0: 41% | 1: 38% | 2: 18% | 3: 3%	1.18 ± 0.96<br>0: 25% | 1: 45% | 2: 18% | 3: 11% | 4: 1%	*** 0.0002<br>** 0.0052
57 Patients With Dyskinesias Evaluation Before & After CSAI Initiation within 24 months	1.13 ± 0.88<br>0: 27% | 1: 38% | 2: 29% | 3: 5%	1.22 ± 0.98<br>0: 24% | 1: 44% | 2: 22% | 3: 9% | 4: 2%	0.4384<br>0.7630
36 Patients With Dyskinesias Evaluation Before & After CSAI Initiation within 12 months	1.13 ± 0.87<br>0: 22% | 1: 50% | 2: 19% | 3: 8%	1.08 ± 0.97<br>0: 31% | 1: 42% | 2: 17% | 3: 11%	0.7055<br>0.6810

Axial: Dysarthria
[Mean ± SD | Breakdown (%)]

(Sub)population	Before CSAI	After CSAI	*p-value* ▲
Overall 142 Patients With Dysarthria Evaluation Before & After CSAI Initiation	0.46 ± 0.64<br>0: 62% | 1: 30% | 2: 8%	0.86 ± 0.88<br>0: 41% | 1: 37% | 2: 18% | 3: 4% | 4: 1%	*** <0.0001<br>*** <0.0001
117 Patients With Dysarthria Evaluation Before & After CSAI Initiation	0.53 ± 0.66<br>0: 56% | 1: 34% | 2: 9%	0.81 ± 0.85<br>0: 43% | 1: 37% | 2: 18% | 3: 2% | 4: 1%	*** 0.0001<br>* 0.0113
54 Patients With Dysarthria Evaluation Before & After CSAI Initiation within 24 months	0.65 ± 0.73<br>0: 50% | 1: 35% | 2: 15%	0.70 ± 0.84<br>0: 52% | 1: 28% | 2: 19% | 3: 2%	0.5254<br>0.8840
35 Patients With Dysarthria Evaluation Before & After CSAI Initiation within 12 months	0.71 ± 0.75<br>0: 46% | 1: 37% | 2: 17%	0.69 ± 0.76<br>0: 49% | 1: 34% | 2: 17%	0.8211<br>0.8510

Axial: Freezing
[Mean ± SD | Breakdown (%)]

(Sub)population	Before CSAI	After CSAI	*p-value* ▲
Overall 144 Patients With Freezing Evaluation Before & After CSAI Initiation	0.46 ± 0.75<br>0: 68% | 1: 19% | 2: 11% | 3: 1%	0.88 ± 1.0<br>0: 46% | 1: 30% | 2: 17% | 3: 5% | 4: 2%	*** <0.0001<br>*** 0.0001
119 Patients With Freezing Evaluation Before & After CSAI Initiation within 60 months	0.54 ± 0.79<br>0: 64% | 1: 22% | 2: 14% | 3: 2%	0.88 ± 1.03<br>0: 47% | 1: 30% | 2: 17% | 3: 5% | 4: 2%	*** 0.0010<br>** 0.0066
55 Patients With Freezing Evaluation Before & After CSAI Initiation within 24 months	0.62 ± 0.87<br>0: 61% | 1: 22% | 2: 15% | 3: 4%	0.73 ± 0.93<br>0: 54% | 1: 28% | 2: 17% | 3: 2% | 4: 2%	0.5005<br>0.4990
34 Patients With Freezing Evaluation Before & After CSAI Initiation within 12 months	0.68 ± 0.91<br>0: 56% | 1: 27% | 2: 12% | 3: 6%	0.85 ± 0.99<br>0: 44% | 1: 35% | 2: 15% | 3: 3% | 4: 3%	0.3739<br>0.3930

▲ *Paired proportions and continuous scores compared using the McNemar's or Wilcoxon signed-rank test, respectively. Exact scores distribution before vs after pump initiation were compared using ordered logistic regression. NA*: Not applicable.

Supplementary Table S2B - Summary Motor Complication & Axial Scores Before vs After CSAI Initiation

Axial: Postural Instability
[Mean ± SD | Breakdown (%)]

(Sub)population	Before CSAI	After CSAI	*p-value* ▲
Overall 142 Patients With Instability Evaluation Before & After CSAI Initiation	0.45 ± 0.70<br>0: 65% | 1: 26% | 2: 8% | 3: 1%	0.88 ± 1.05<br>0: 47% | 1: 30% | 2: 13% | 3: 8% | 4: 2%	*** <0.0001<br>0.0005
117 Patients With Instability Evaluation Before & After CSAI Initiation within 60 months	0.53 ± 0.74<br>0: 60% | 1: 29% | 2: 9% | 3: 2%	0.79 ± 0.96<br>0: 49% | 1: 32% | 2: 12% | 3: 7% | 4: 1%	*** 0.0004<br>0.0421
54 Patients With Instability Evaluation Before & After CSAI Initiation within 24 months	0.70 ± 0.83<br>0: 50% | 1: 33% | 2: 13% | 3: 4%	0.72 ± 0.96<br>0: 54% | 1: 30% | 2: 7% | 3: 9%	0.82960<br>0.8310
34 Patients With Instability Evaluation Before & After CSAI Initiation within 12 months	0.76 ± 0.89<br>0: 47% | 1: 35% | 2: 12% | 3: 6%	0.76 ± 0.96<br>0: 50% | 1: 32% | 2: 9% | 3: 9%	1.0000<br>0.8820

Axial: Postural Deformation
[Mean ± SD | Median (Q1-Q3)]

(Sub)population	Before CSAI	After CSAI	*p-value* ▲
Overall 136 Patients With Deformation Evaluation Before & CSAI Pump Initiation	0.35 ± 0.70<br>0: 75% | 1: 18% | 2: 4% | 3: 1% | 4: 1%	0.76 ± 0.94<br>0: 49% | 1: 34% | 2: 10% | 3: 5% | 4: 1%	*** 0.0001<br>*** <0.0001
117 Patients With Deformation Evaluation Before & After CSAI Initiation within 60 months	0.40 ± 0.74<br>0: 71% | 1: 21% | 2: 2% | 3: 2% | 4: 1%	0.75 ± 0.93<br>0: 49% | 1: 35% | 2: 10% | 3: 4% | 4: 2%	*** <0.0001<br>*** 0.0001
51 Patients With Deformation Evaluation Before & After CSAI Initiation within 24 months	0.57 ± 0.92<br>0: 63% | 1: 26% | 2: 6% | 3: 4% | 4: 2%	0.69 ± 0.97<br>0: 55% | 1: 31% | 2: 6% | 3: 6% | 4: 1%	0.25990<br>0.4420
31 Patients With Deformation Evaluation Before & After CSAI Initiation within 12 months	0.61 ± 1.05<br>0: 65% | 1: 23% | 2: 3% | 3: 6% | 4: 3%	0.81 ± 1.05<br>0: 48% | 1: 36% | 2: 6% | 3: 6% | 3: 3%	* 0.0411<br>0.2560

Axial: Swallowing Problems
[Mean ± SD | Breakdown (%)]

(Sub)population	Before CSAI	After CSAI	*p-value* ▲
Overall 141 Patients With Swallowing Evaluation Before & After CSAI Initiation	0.13 ± 0.38<br>0: 89% | 1: 10% | 2: 1%	0.27 ± 0.57<br>0: 79% | 1: 14% | 2: 6%	** 0.0074<br>* 0.0295
116 Patients With Swallowing Evaluation Before & After CSAI Initiation	0.15 ± 0.40<br>0: 87% | 1: 11% | 2: 2%	0.26 ± 0.56<br>0: 80% | 1: 14% | 2: 6%	* 0.046<br>0.1350
51 Patients With Swallowing Evaluation Before & After CSAI Initiation within 24 months	0.20 ± 0.45<br>0: 82% | 1: 16% | 2: 2%	0.25 ± 0.52<br>0: 78% | 1: 18% | 2: 4%	0.3741<br>0.5960
33 Patients With Swallowing Evaluation Before & After CSAI Initiation within 12 months	0.21 ± 0.48<br>0: 82% | 1: 15% | 2: 3%	0.21 ± 0.48<br>0: 82% | 1: 15% | 2: 3%	1.0000<br>1.0000

Axial: Falls
[Breakdown (%)]

(Sub)population	Before CSAI	After CSAI	*p-value* ▲
Overall 144 Patients With Freezing Evaluation Before & After CSAI Initiation	Non: 68% | Yes: 19%	Non: 46% | Yes: 30%	0.0543
114 Patients With Freezing Evaluation Before & After CSAI Initiation within 60 months	Non: 76% | Yes: 24%	Non: 67% | Yes: 33%	0.0543
51 Patients With Freezing Evaluation Before & After CSAI Initiation within 24 months	Non: 69% | Yes: 31%	Non: 67% | Yes: 33%	1.000
33 Patients With Freezing Evaluation Before & After CSAI Initiation within 12 months	Non: 61% | Yes: 39%	Non: 67% | Yes: 33%	0.7237

▲ *Paired proportions and continuous scores compared using the McNemar's or Wilcoxon signed-rank test, respectively. Exact scores distribution before vs after pump initiation were compared using ordered logistic regression. NA*: Not applicable.

Supplementary Table S2C- Summary Autonomic Scores Before vs After CSAI Initiation

Dysautonomia: Hypotension
[Mean ± SD | Breakdown (%)]

(Sub)population	Before CSAI	After CSAI	*p-value* ▲
Overall 122 Patients With Hypotension Evaluation Before & After CSAI Initiation	0.20 ± 0.50<br>0: 83% | 1: 15% | 2: 2% | 3: 1%	0.35 ± 0.71<br>0: 75% | 1: 19% | 2: 4% | 3: 2% | 4: 1%	* 0.0196<br>0.1000
118 Patients With Hypotension Evaluation Before & After CSAI Initiation within 60 months	0.21 ± 0.50<br>0: 82% | 1: 15% | 2: 2% | 3: 1%	0.36 ± 0.72<br>0: 75% | 1: 20% | 2: 4% | 3: 2% | 4: 1%	* 0.0196<br>0.0979
53 Patients With Hypotension Evaluation Before & After CSAI Initiation within 24 months	0.30 ± 0.61<br>0: 76% | 1: 21% | 2: 2% | 3: 2%	0.26 ± 0.56<br>0: 77% | 1: 21% | 2: 2%	0.6078<br>0.7870
36 Patients With Hypotension Evaluation Before & After CSAI Initiation within 12 months	0.32 ± 0.65<br>0: 74% | 1: 23% | 2: 3%	0.26 ± 0.63<br>0: 81% | 1: 16% | 2: 3%	0.4237<br>0.5590

Dysautonomia: Digestive problems
[Mean ± SD | Breakdown (%)]

(Sub)population	Before CSAI	After CSAI	*p-value* ▲
Overall 138 Patients With Digestive Evaluation Before & After CSAI Initiation	0.49 ± 0.76<br>0: 65% | 1: 23% | 2: 9% | 3: 2%	0.65 ± 0.86<br>0: 57% | 1: 25% | 2: 17% | 3: 1% | 4: 1%	* 0.0139<br>0.0968
114 Patients With Digestive Evaluation Before & After CSAI Initiation within 60 months	0.57 ± 0.80<br>0: 60% | 1: 26% | 2: 11% | 3: 3%	0.72 ± 0.89<br>0: 53% | 1: 26% | 2: 18% | 3: 2% | 4: 1%	0.0525<br>0.2040
54 Patients With Digestive Evaluation Before & After CSAI Initiation within 24 months	0.76 ± 0.95<br>0: 54% | 1: 22% | 2: 19% | 3: 6%	0.89 ± 0.98<br>0: 44% | 1: 30% | 2: 20% | 3: 4% | 4: 2%	0.3102<br>0.4430
34 Patients With Digestive Evaluation Before & After CSAI Initiation within 12 months	0.79 ± 0.98<br>0: 50% | 1: 29% | 2: 12% | 3: 9%	1.0 ± 1.04<br>0: 38% | 1: 35% | 2: 18% | 3: 6% | 4: 3%	0.2379<br>0.3590

Dysautonomia: Urinary
[Mean ± SD | Breakdown (%)]

(Sub)population	Before CSAI	After CSAI	*p-value* ▲
Overall 139 Patients With Urinary Evaluation Before & After CSAI Initiation	0.34 ± 0.60<br>0: 71% | 1: 24% | 2: 4% | 3: 1%	0.75 ± 0.98<br>0: 54% | 1: 25% | 2: 14% | 3: 5% | 4: 1%	*** <0.0001<br>*** 0.0004
114 Patients With Urinary Evaluation Before & After CSAI Initiation	0.36 ± 0.63<br>0: 71% | 1: 23% | 2: 5% | 3: 1%	0.69 ± 0.91<br>0: 56% | 1: 24% | 2: 15% | 3: 5%	*** 0.0001<br>** 0.0056
53 Patients With Urinary Evaluation Before & After CSAI Initiation within 24 months	0.34 ± 0.68<br>0: 74% | 1: 19% | 2: 6% | 3: 2%	0.47 ± 0.77<br>0: 68% | 1: 19% | 2: 11% | 3: 2%	0.1581<br>0.4610
34 Patients With Urinary Evaluation Before & After CSAI Initiation within 12 months	0.35 ± 0.69<br>0: 74% | 1: 21% | 2: 3% | 3: 3%	0.44 ± 0.79<br>0: 71% | 1: 18% | 2: 9% | 3: 3%	0.4290<br>0.7060

▲ *Paired continuous scores compared using the Wilcoxon signed-rank test, respectively. Exact scores distribution before vs after pump initiation were compared using ordered logistic regression. NA*: Not applicable.

Supplementary Table S2D - Summary Psychiatric Before vs After CSAI Initiation

Psychiatric: Apathy [Mean ± SD | Breakdown (%)]			
(Sub)population	Before CSAI	After CSAI	*p-value* ▲
Overall 145 Patients With Apathy Evaluation Before & After CSAI Initiation	0.12 ± 0.36<br>0: 90% | 1: 9% | 2: 1%	0.31 ± 0.67<br>0: 79% | 1: 14% | 2: 6% | 3: 2%	*** 0.0006139<br>0.00852
120 Patients With Apathy Evaluation Before & After CSAI Initiation within 60 months	0.13 ± 0.38<br>0: 89% | 1: 9% | 2: 2%	0.27 ± 0.58<br>0: 80% | 1: 13% | 2: 7%	** 0.005308<br>0.0439
57 Patients With Apathy Evaluation Before & After CSAI Initiation within 24 months	0.18 ± 0.47<br>0: 86% | 1: 11% | 2: 4%	0.23 ± 0.54<br>0: 83% | 1: 12% | 2: 5%	0.407<br>0.597
35 Patients With Apathy Evaluation Before & After CSAI Initiation within 12 months	0.14 ± 0.43<br>0: 89% | 1: 9% | 2: 3%	0.20 ± 0.53<br>0: 86% | 1: 9% | 2: 6%	0.5862<br>0.699

Psychiatric: Depression [Mean ± SD | Breakdown (%)]			
(Sub)population	Before CSAI	After CSAI	*p-value* ▲
Overall 146 Patients With Depression Evaluation Before & After CSAI Initiation	0.35 ± 0.57<br>0: 70% | 1: 25% | 2: 5%	0.67 ± 0.86<br>0: 55% | 1: 27% | 2: 15% | 3: 3%	*** 0.00001129<br>0.00143
121 Patients With Depression Evaluation Before & After CSAI Initiation within 60 months	0.36 ± 0.59<br>0: 69% | 1: 25% | 2: 6%	0.60 ± 0.81<br>0: 59% | 1: 26% | 2: 13% | 3: 2%	** 0.001255<br>0.0354
57 Patients With Depression Evaluation Before & After CSAI Initiation within 24 months	0.49 ± 0.68<br>0: 61% | 1: 28% | 2: 11%	0.61 ± 0.82<br>0: 56% | 1: 30% | 2: 11% | 3: 4%	0.1562<br>0.499
36 Patients With Depression Evaluation Before & After CSAI Initiation within 12 months	0.56 ± 0.69<br>0: 56% | 1: 33% | 2: 11%	0.67 ± 0.93<br>0: 58% | 1: 22% | 2: 14% | 3: 6%	0.3075<br>0.878

Psychiatric: Anxiety [Mean ± SD | Breakdown (%)]			
(Sub)population	Before CSAI	After CSAI	*p-value* ▲
Overall 147 Patients With Anxiety Evaluation Before & After CSAI Initiation	0.50 ± 0.73<br>0: 61% | 1: 31% | 2: 6% | 3: 1% | 4: 1%	0.86 ± 0.98<br>0: 46% | 1: 31% | 2: 18% | 3: 4% | 4: 2%	*** <0.0001<br>0.0338
122 Patients With Anxiety Evaluation Before & After CSAI Initiation within 60 months	0.55 ± 0.77<br>0: 58% | 1: 32% | 2: 7% | 3: 2% | 4: 1%	0.83 ± 0.99<br>0: 48% | 1: 29% | 2: 17% | 3: 3% | 4: 2%	*** 0.0002<br>* 0.0320
58 Patients With Anxiety Evaluation Before & After CSAI Initiation within 24 months	0.77 ± 0.94<br>0: 50% | 1: 31% | 2: 14% | 3: 3% | 4: 2%	0.90 ± 1.05<br>0: 45% | 1: 33% | 2: 14% | 3: 5% | 4: 3%	0.1902<br>0.5180
36 Patients With Anxiety Evaluation Before & After CSAI Initiation within 12 months	0.83 ± 1.03<br>0: 47% | 1: 33% | 2: 11% | 3: 6% | 4: 3%	0.94 ± 1.09<br>0: 42% | 1: 36% | 2: 14% | 3: 3% | 3: 6%	0.3785<br>0.6390

Psychiatric: Hallucinations [Mean ± SD | Breakdown (%)]			
(Sub)population	Before CSAI	After CSAI	*p-value* ▲
Overall 143 Patients With Hallucinations Evaluation Before & After CSAI Initiation	0.08 ± 0.35<br>0: 94% | 1: 4% | 2: 2%	0.23 ± 0.51<br>0: 81% | 1: 15% | 2: 4%	*** 0.0010<br>** 0.0024
119 Patients With Hallucinations Evaluation Before & After CSAI Initiation	0.10 ± 0.38<br>0: 92% | 1: 5% | 2: 3%	0.25 ± 0.52<br>0: 79% | 1: 17% | 2: 4%	** 0.0024<br>** 0.0050
57 Patients With Hallucinations Evaluation Before & After CSAI Initiation within 24 months	0.16 ± 0.49<br>0: 90% | 1: 5% | 2: 5%	0.30 ± 0.60<br>0: 77% | 1: 16% | 2: 7%	0.0709<br>0.0981
35 Patients With Hallucinations Evaluation Before & After CSAI Initiation within 12 months	0.14 ± 0.49<br>0: 91% | 1: 3% | 2: 6%	0.26 ± 0.51<br>0: 77% | 1: 20% | 2: 3%	0.2755<br>0.1410

▲ *Paired continuous scores compared using the Wilcoxon signed-rank test, respectively. Exact scores distribution before vs after pump initiation were compared using ordered logistic regression. NA*: Not applicable.

Supplementary Table S2E - Other Summary Signs|Symptoms Before vs After CSAI Initiation

Somnolence
[Mean ± SD | Breakdown (%)]

(Sub)population	Before CSAI	After CSAI	*p-value* ▲
Overall 144 Patients With Somnolence Evaluation Before & After CSAI Initiation	0.51 ± 0.69<br>0: 59% | 1: 31% | 2: 9% | 3: 1%	0.83 ± 0.82<br>0: 40% | 1: 40% | 2: 16% | 3: 3%	*** 0.0001<br>*** 0.0007
119 Patients With Somnolence Evaluation Before & After CSAI Initiation within 60 months	0.55 ± 0.72<br>0: 58% | 1: 30% | 2: 11% | 3: 1%	0.78 ± 0.78<br>0: 41% | 1: 42% | 2: 14% | 3: 3%	** 0.0046<br>* 0.0123
56 Patients With Somnolence Evaluation Before & After CSAI Initiation within 24 months	0.61 ± 0.78<br>0: 55% | 1: 30% | 2: 13% | 3: 2%	0.70 ± 0.76<br>0: 46% | 1: 39% | 2: 13% | 3: 2%	0.4735<br>0.4400
36 Patients With Somnolence Evaluation Before & After CSAI Initiation within 12 months	0.61 ± 0.77<br>0: 53% | 1: 36% | 2: 8% | 3: 3%	0.56 ± 0.69<br>0: 56% | 1: 33% | 2: 11%	0.6600<br>0.8190

Psychiatric: Cognitive Impairment
[Mean ± SD | Breakdown (%)]

(Sub)population	Before CSAI	After CSAI	*p-value* ▲
Overall 137 Patients With Cognitive Impairment Evaluation Before & After CSAI Initiation	0.36 ± 0.70<br>0: 75% | 1: 18% | 2: 7% | 3: 1% | 4: 1%	0.80 ± 0.91<br>0: 47% | 1: 30% | 2: 20% | 3: 1% | 4: 1%	*** 0.0001<br>*** 0.0001
113 Patients With Cognitive Impairment Evaluation Before & After CSAI Initiation within 60 months	0.42 ± 0.75<br>0: 70% | 1: 20% | 2: 8% | 3: 1% | 4: 1%	0.69 ± 0.80<br>0: 51% | 1: 29% | 2: 19% | 3: 1%	*** 0.0004<br>** 0.0039
52 Patients With Cognitive Impairment Evaluation Before & After CSAI Initiation within 24 months	0.60 ± 0.93<br>0: 64% | 1: 19% | 2: 14% | 3: 2% | 4: 2%	0.54 ± 0.75<br>0: 62% | 1: 23% | 2: 15%	0.6444<br>0.9760
35 Patients With Cognitive Impairment Evaluation Before & After CSAI Initiation within 12 months	0.60 ± 0.98<br>0: 63% | 1: 23% | 2: 9% | 3: 3% | 4: 3%	0.51 ± 0.74<br>0: 63% | 1: 23% | 2: 14%	0.6078<br>0.9450

▲ *Paired continuous scores compared using the Wilcoxon signed-rank test, respectively. Exact scores distribution before vs after pump initiation were compared using ordered logistic regression. NA*: Not applicable.
